# Molecular Evolutionary Analysis of the Influenza A(H1N1)pdm, May–September, 2009: Temporal and Spatial Spreading Profile of the Viruses in Japan

**DOI:** 10.1371/journal.pone.0011057

**Published:** 2010-06-10

**Authors:** Teiichiro Shiino, Nobuhiko Okabe, Yoshinori Yasui, Tomimasa Sunagawa, Makoto Ujike, Masatsugu Obuchi, Noriko Kishida, Hong Xu, Emi Takashita, Akane Anraku, Reiko Ito, Teruko Doi, Miho Ejima, Hiromi Sugawara, Hiroshi Horikawa, Shuji Yamazaki, Yumiko Kato, Akio Oguchi, Nobuyuki Fujita, Takato Odagiri, Masato Tashiro, Haruo Watanabe

**Affiliations:** 1 Infectious Diseases Surveillance Center, National Institute of Infectious Diseases, Tokyo, Japan; 2 Influenza Virus Research Center, National Institute of Infectious Diseases, Tokyo, Japan; 3 Genome Analysis Center, Department of Biotechnology, National Institute of Technology and Evaluation, Tokyo, Japan; Institute of Infectious Disease and Molecular Medicine, South Africa

## Abstract

**Background:**

In March 2009, pandemic influenza A(H1N1) (A(H1N1)pdm) emerged in Mexico and the United States. In Japan, since the first outbreak of A(H1N1)pdm in Osaka and Hyogo Prefectures occurred in the middle of May 2009, the virus had spread over 16 of 47 prefectures as of June 4, 2009.

**Methods/Principal Findings:**

We analyzed all-segment concatenated genome sequences of 75 isolates of A(H1N1)pdm viruses in Japan, and compared them with 163 full-genome sequences in the world. Two analyzing methods, distance-based and Bayesian coalescent MCMC inferences were adopted to elucidate an evolutionary relationship of the viruses in the world and Japan. Regardless of the method, the viruses in the world were classified into four distinct clusters with a few exceptions. Cluster 1 was originated earlier than cluster 2, while cluster 2 was more widely spread around the world. The other two clusters (clusters 1.2 and 1.3) were suggested to be distinct reassortants with different types of segment assortments. The viruses in Japan seemed to be a multiple origin, which were derived from approximately 28 transported cases. Twelve cases were associated with monophyletic groups consisting of Japanese viruses, which were referred to as micro-clade. While most of the micro-clades belonged to the cluster 2, the clade of the first cases of infection in Japan originated from cluster 1.2. Micro-clades of Osaka/Kobe and the Fukuoka cases, both of which were school-wide outbreaks, were eradicated. Time of most recent common ancestor (tMRCA) for each micro-clade demonstrated that some distinct viruses were transmitted in Japan between late May and early June, 2009, and appeared to spread nation-wide throughout summer.

**Conclusions:**

Our results suggest that many viruses were transmitted from abroad in late May 2009 irrespective of preventive actions against the pandemic influenza, and that the influenza A(H1N1)pdm had become a pandemic stage in June 2009 in Japan.

## Introduction

Since pandemic influenza A(H1N1) (A(H1N1)pdm) emerged in Mexico and the United States (US) in March 2009 [1], [2], human-to-human transmission enabled rapid, worldwide spread of the virus during the first few weeks of the pandemic. Over 29,000 cases and 145 deaths were reported in over 74 countries as of June 12, 2009 [3], prompting the World Health Organization (WHO) to raise the influenza pandemic alert level to 6. Since April 28, 2009, the Ministry of Health, Labour and Welfare in Japan required febrile travelers returning from affected areas to be screened at the point of entry [4]. A conventional and/or real-time RT-PCR test for A(H1N1)pdm virus, developed at the National Institute of Infectious Diseases, was launched on May 4, 2009, by the quarantine stations and the prefectural and the municipal public health institutes in Japan. The first confirmed cases were detected at the Narita International airport quarantine station on May 9, 2009 at four travelers returned from Canada [5]. The Quarantine Law and the Pandemic Influenza Preparedness Action Plan of the Japanese Government [5] requires all patients to be isolated in a designated hospital for seven days. One student who had attended a high school in Ibaraki City in Osaka Prefecture and four others who had attended a high school in Kobe City in Hyogo Prefecture, were confirmed to have A(H1N1)pdm virus infection on May 16, 2009 [5]. School-wide outbreaks of influenza A were reported over the next few days, and all schools in Osaka Prefecture and Kobe City were closed for either one or two weeks starting from May 16, 2009. Although the number of newly confirmed cases in the schools decreased after the school closures, a few cases that had an epidemiological link to a traveler from those 2 prefectures were reported in the peripheral areas [5]. On May 22, the Japanese government revised its Action Plan to relax quarantine, school closure, and medical service regulations considering circumstances like local infection sites. According to this revision, the febrile screening at airport-quarantines was expired. After the Osaka/Kobe outbreak, some sporadic cases occurred also in other prefectures. A total of 401 cases from 16 of 47 prefectures in Japan were reported as of June 4, 2009. After WHO raised the influenza pandemic alert level to phase 6 on June 11,2009, Japan faced two more school-based outbreaks of A(H1N1)pdm in Fukuoka [6] and Chiba prefectures [7]. The Japanese government revised its Action Plan again after these outbreaks, switching to the sentinel surveillance system [8] that was a same manner for category V infectious diseases in the Infection Diseases Control Low [9].

The genetic composition of the A(H1N1)pdm virus was available early in the pandemic [10], and more than 6000 gene sequences from 3600 individuals were deposited in the GenBank database by the end of September 2009. The virus was found to have a genome with an extremely high evolutionary rate (3.66×10^−3^ substitution per site per year) [11]. Accurate analysis of the viral genome-wide sequence may help us infer the evolutionary development over a very small time-scale, (since the beginning of the pandemic A(H1N1)). The phylogenetic relationship of the A(H1N1)pdm viruses over the first pandemic wave was recently analyzed using a concatenated sequence of six viral segments [12]. This study reported the circulation of two distinct genetic groups (clusters) during the pandemic phase. They also identified three sub-clusters in cluster 1. All the viruses isolated from the Osaka/Kobe outbreak case [5] belonged to the small sub-cluster 1.2, which had a cluster 2-like nucleoprotein [12]. Viruses from sub-cluster 1.2, and from sub-cluster 1.3, may be reassortants between two major clusters [12].

As of September 30, the GenBank/NIAID database has started providing the full-genome sequences of 452 viruses, of which 75 viruses were derived from Japan. Some of these viruses were available for study of the epidemiological linkage of the infected individuals in our previous study, but many, especially after enforcement of sentinel surveillance, were not. A phylogenetic inference of full-genome sequences using a time-based Bayesian coalescent Markov Monte Carlo (Bayesian MCMC) method may describe an important evolutionary process underlying the A(H1N1)pdm outbreak in Japan. Our results support the current epidemiological information about the 2009 influenza A(H1N1) pandemic in Japan.

## Results

**Figure 1:**
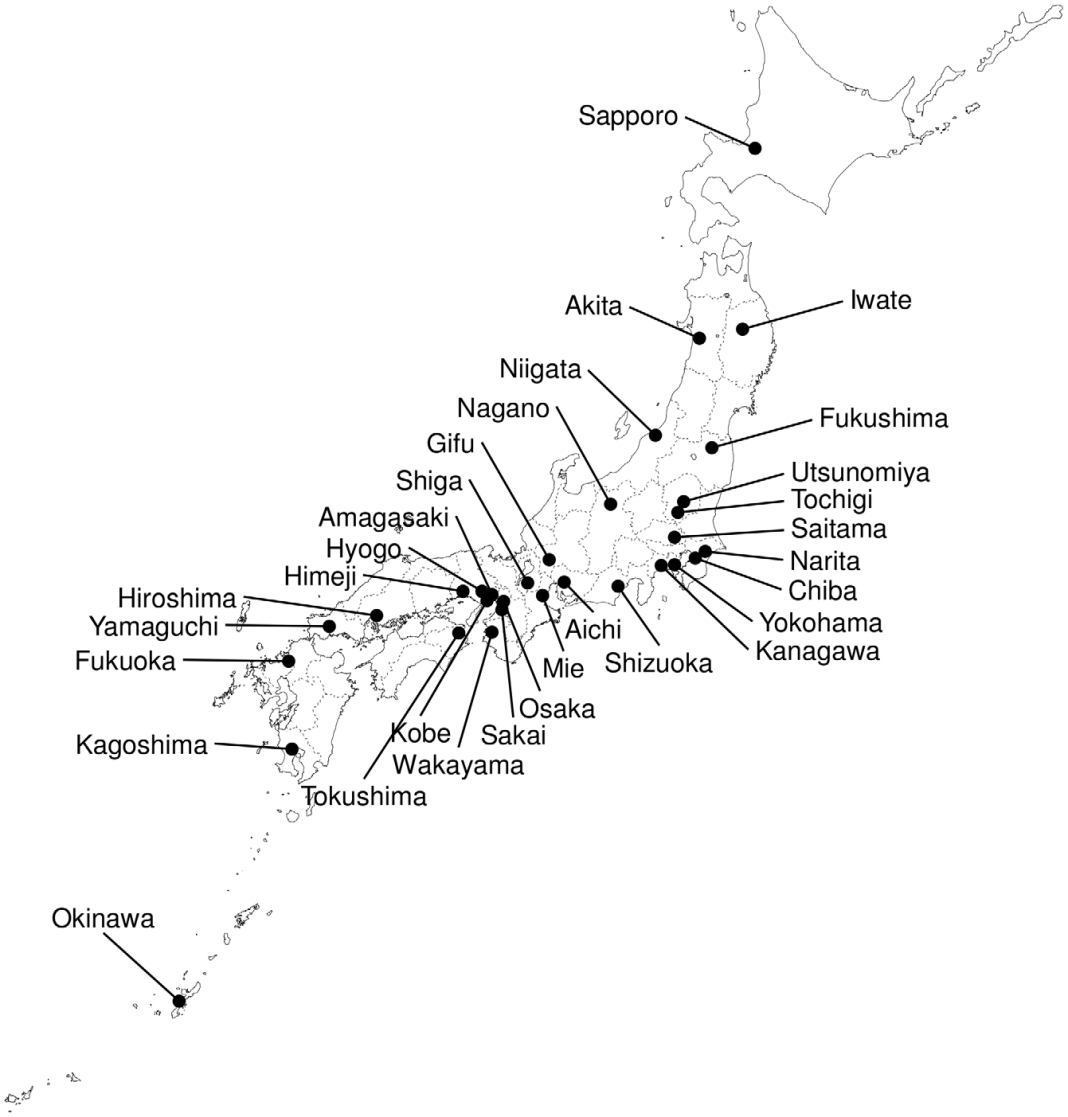
Geographical distribution of virus isolates of influenza A(H1N1)pdm in Japan.

Since the first outbreak of the A(H1N1)pdm infection occurred in the Osaka/Kobe district of Japan in May, the virus infection gradually extended nation-wide, from the beginning of June. Field epidemiological investigations reported four other outbreaks cases during this period (Table S1). The virus had colonized all prefectures in Japan by the middle of July. Then Japan abandoned the practice of reporting every case of pandemic virus infections. We collected viruses (Figure 1) isolated from hospitalized patients between May 8, 2009, and September 22, 2009 and sequenced the full-length genome of 8 segments of the 74 viruses. We also found a full-length sequence that was apparently derived from Japan (A/Japan/PR1070/2009(H1N1)) in GenBank genome database. Consequently, we obtained 75 full-length sequences of Japanese isolates of A(H1N1)pdm.

**Figure 2:**
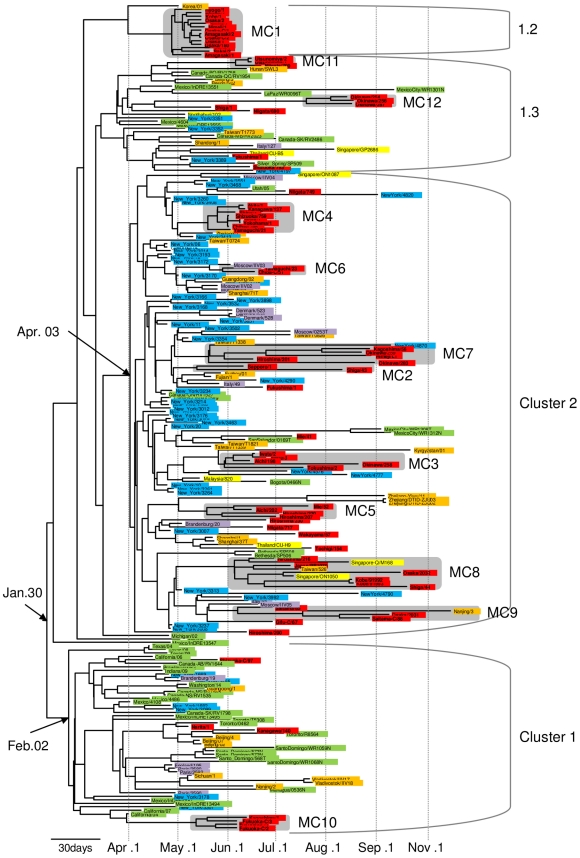
Bayesian coalescence phylogeny of influenza A(H1N1)pdm full-genome sequences from the world. The branch length of the phylogeny is in units of time. The scale bar indicating 30 days of the branch length is drawn at the bottom of the tree. The clusters described in the previous report [12] are annotated by brackets on the right of the tree. The sequences from Japan are colored with red. The sequences from Central-East Asia except for Japan, South-East Asia, Europe, New York, and Central-North America except for New York are colored with orange, yellow, purple, blue and green, respectively. Shadows on the terminal lineages show Japanese micro-clades. Date with arrows pointing out a node of the tree shows tMRCA of the monophyletic group. MC represents micro-clade.

**Figure 3:**
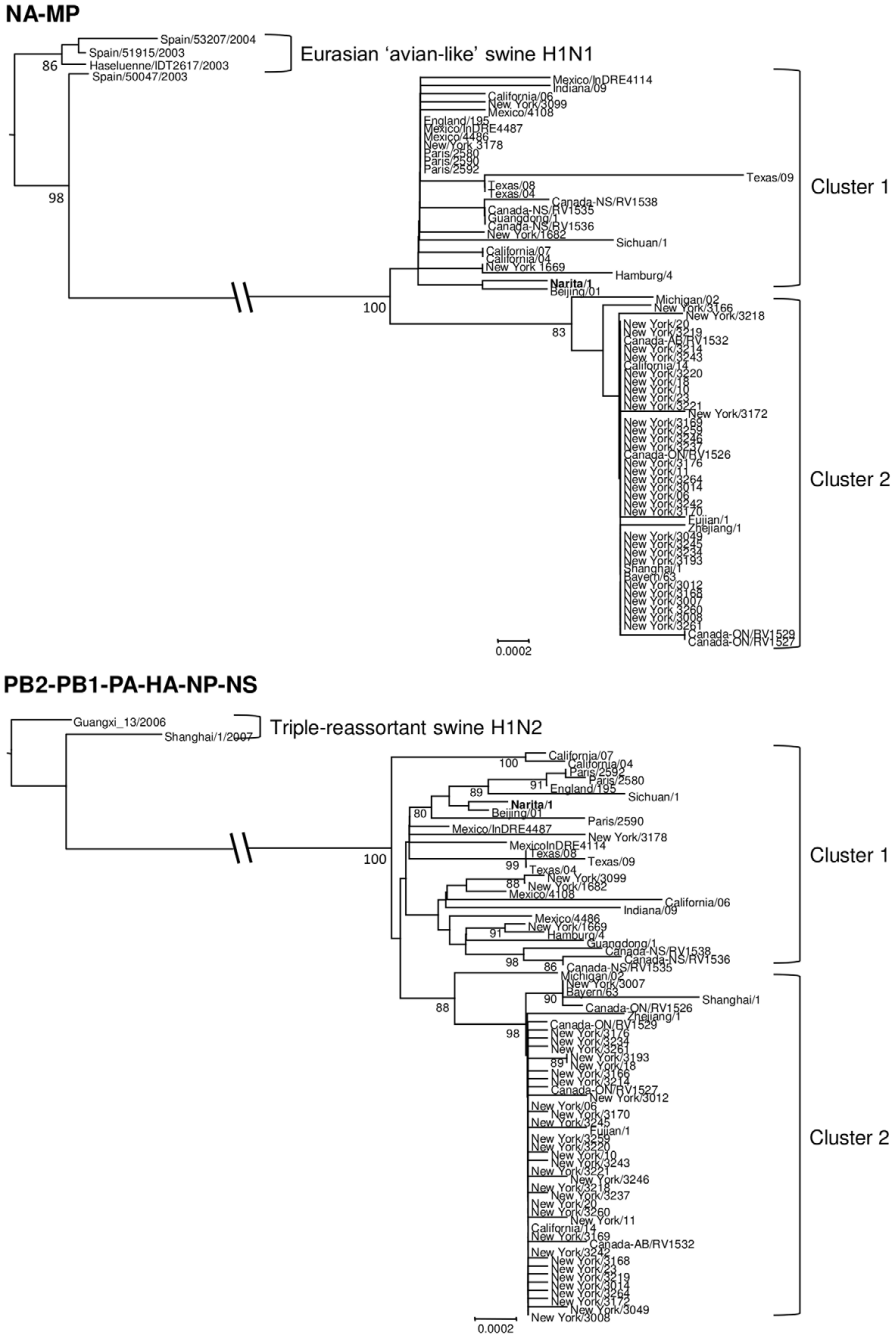
Distance-based neighbor joining phylogeny of influenza A(H1N1)pdm in early phase of the pandemic with particular swine influenza strains. The upper panel shows the phylogeny using NA-MP that was derived from Eurasian avian-like swine H1N1 virus. The lower panel shows the phylogeny using PB2-PB1-PA-HA-NP-NS that was derived from the triple reassortant swine H1N2/3. Bold name shows Narita/1 which was the first confirmed case in Japan. Both trees were re-rooted with swine influenza strains. The scale bar in the units of the number of base substitutions per site is drawn at the bottom of each tree.

We computed the genetic diversity of our isolates in Japan with that of isolates in the world. The entire nucleotide diversity in a total of 238 sequences collected from the world including Japan and 75 Japanese isolates were 0.00164 and 0.00183, respectively, which seem to be identical (Table S2). A Bayesian coalescent-based phylogenetic inference demonstrated the worldwide circulation of four distinct clusters with a few exceptions (Figure 2), which was consistent with the result by the distance-based phylogenetic inference (Figure S1). Divergence topology of the phylogeny was similar to a previously reported Neighbor-Joining tree [12] whose cluster 1.1 corresponded to our cluster 1. The cluster 2 in both studies was identical. We applied a Bayesian relaxed molecular clock method to the phylogeny and estimated the mean evolutionary rate of the full-length genome of A(H1N1)pdm to be 4.0×10^−3^ substitutions per site per year (95% probability density ranged 3.6×10^−3^ to 4.4×10^−3^: Table 1). The estimated mean coefficient of variation was 0.34, indicating substantial heterogeneity in the evolutionary rate in viral lineage. Tajima's D statistic for full-length sequence alignment was −2.806 (Table 2), demonstrating a weak purifying selection during the evolutionary process of A(H1N1)pdm in 2009. The Bayesian chronological phylogeny also showed that cluster 1 had an older ancestor than cluster 2. Based on the estimates of the evolutionary rate, tMRCA of cluster 1 was dated to February 2, 2009 (range from January 11 to February. 26: Table 1), whereas that of the cluster 2 was April 3, 2009 (range from March 25 to April 9: Table 1). Mean root height, i.e. tMRCA of whole samples, was estimated to January 30, 2009 (range from January 7 to February 18, 2009: Table 1). We used pre-reassorted ancestral sequences as an outgroup to show that the common ancestor of A(H1N1)pdm viruses belonged to cluster 1 (Figure 3). Cluster 2 formed a monophyletic group, while cluster 1 was paraphyletic. Viruses in the cluster 2 were more widely distributed when compared with cluster 1, and were found in more recently infected patients (Figure 2).

**Table 1:** Evolutionary parameters of A(H1N1)pdm viruses based on the full-genome sequence.

	Mean	95% HPD^a^ confidence interval	
Population size	3242.88	1717.59	5323.27
Growth rate	0.05	0.04	0.07
Mean evolutionary rate	4.01×10^−3^	3.59×10^−3^	4.44×10^−3^
Coeff.of.Variates	0.3434	0.00	0.65
Likelihood	−2.98×10^+4^	−2.99×10^+4^	−2.98×10^+4^
Root height	30-Jan-09	18-Feb-09	7-Jan-09
tMRCA of MC1^b^	22-Apr-09	1-May-09	11-Apr-09
tMRCA of MC2^b^	15-May-09	1-Jun-09	5-May-09
tMRCA of MC3^b^	16-May-09	29-May-09	2-May-09
tMRCA of MC4^b^	17-May-09	26-May-09	7-May-09
tMRCA of MC5^b^	19-May-09	2-Jun-09	4-May-09
tMRCA of MC6^b^	25-May-09	9-Jun-09	8-May-09
tMRCA of MC7^b^	25-May-09	7-Jun-09	11-May-09
tMRCA of MC8^b^	29-May-09	13-Jun-09	12-May-09
tMRCA of MC9^b^	9-Jun-09	29-Jun-09	21-May-09
tMRCA of MC10^b^	21-May-09	1-Jun-09	8-May-09
tMRCA of MC11^b^	5-Jun-09	13-Jun-09	26-May-09
tMRCA of MC12^b^	24-Jul-09	7-Aug-09	8-Jul-09
tMRCA of Cluster 1	2-Feb-09	26-Feb-09	11-Jan-09
tMRAC of Cluster 2	3-Apr-09	9-Apr-09	25-Mar-09

a HPD represents highest posterior density.

b MC represents micro-clade.

**Table 2:** Results from Tajima's Neutrality Test.

m	S	p_s_	Θ	π	D
236	1028	0.083232	0.013783	0.001637	−2.80608

The abbreviations used are as follows: m  =  number of sites, S  =  Number of segregating sites, ps  =  S/m, Θ  =  ps/a1, and π  =  nucleotide diversity. D is the Tajima test statistic.

Other than clusters 1 and 2, our phylogenetic analysis showed two relatively small clusters, which included members of cluster 1.2 and 1.3 from a previous study [12]. Cluster 1.2 contains the viruses from Osaka/Kobe outbreak and one Korean strain, whereas cluster 1.3 contains 10 viruses from various geographical areas and periods in Japan. The phylogenetic analysis (Figure 2, S1) showed that these clusters diverged before the emergence of a common ancestor of the major clusters. Viruses belonging to these clusters were previously suggested to be a reassortant between two major clusters [12]. We examined a cladistical discordance among the segments of these viral genomes by comparing neighbor-joining trees of each segment (Figure S2). Our data showed that viruses in the cluster 1.2 were clearly composed of HA, MP and NS segments from the cluster 1 and the NP segment from the cluster 2 (Table 3). We also showed that viruses in the cluster 1.3 had a different structure when compared with the cluster 1.2. The MP and NS segments in the cluster 1.3 were derived from cluster 1 while the HA, NP and NA segments were derived from the cluster 2. The NA segment of the cluster 1.2 had an interesting sequence which seemed to be an intermediate of the clusters 1 and 2. The origin of other segments in the reassortant clusters was not determined because of an insufficient accumulation of nucleotide substitutions among these genes.

**Table 3:** Phylogenetically inferred cluster of each segment of the A(H1N1)pdm viruses in Japan.

Strain	whole	PB2	PB1	PA	HA	NP	NA	MP	NS
Tokushima/1	1.3	1	ND^a^	ND^a^	2	2	2	1	1
Shizuoka/793	1.3	1	ND	ND	2	2	2	1	1
Niigata/690	1.3	ND^a^	ND	ND	2	2	2	1	1
Okinawa/254	1.3	ND^a^	ND	ND	2	2	2	1	1
Okinawa/254	1.3	ND^a^	ND	ND	2	2	2	1	1
Okinawa/257	1.3	ND^a^	ND	ND	2	2	2	1	1
Shiga/1	1.3	ND^a^	ND	1	2	2	2	1	U
Utsunomiya/1	1.3	ND^a^	ND	ND	2	2	2	1	1
Utsunomiya/2	1.3	ND^a^	ND	ND	2	2	2	1	1
Niigata/700	1.3	ND^a^	ND	ND	2	2	2	1	1
Sakai/1	1.2	ND^a^	ND	ND	1	2	U	1	1
Sakai/2	1.2	ND^a^	ND	ND	1	2	U	1	1
Amagasaki/1	1.2	ND^a^	ND	ND	1	2	U	1	1
Amagasaki/2	1.2	ND^a^	ND	ND	1	2	U	1	1
Kobe/1	1.2	ND^a^	ND	ND	1	2	U	1	1
Osaka/1	1.2	ND^a^	ND	ND	1	2	U	1	1
Osaka/2	1.2	ND^a^	ND	ND	1	2	U	1	1
Osaka-C/1	1.2	ND^a^	ND	ND	1	2	U	1	1
Osaka-C/2	1.2	ND^a^	ND	ND	1	2	U	1	1
Osaka/180	1.2	ND^a^	ND	ND	1	2	2	1	1
Himeji/1	1.2	ND^a^	ND	ND	1	2	U	1	1
Shiga/2	1.2	ND^a^	ND	ND	1	2	U	1	1
Hyogo/1	1.2	ND^a^	ND	ND	1	2	U	1	1
Hyogo/2	1.2	ND^a^	ND	ND	1	2	U	1	1
Korea/1	1.2	ND^a^	ND	ND	1	2	U	1	1

a ND represents cannot determine a belonging cluster because of few divergences.

We distinguished Japanese viruses into some groups that were estimated to be derived from a single common transported case, using two full-length phylogenies according to a given criteria (see Materials and Methods). Such a group was referred to as a micro-clade. Fifty nine of the 75 strains isolated in Japan were separated into the 12 micro-clades in the full-length phylogeny (Figure 2 and S1). Mean evolutionary diversity within micro-clades was 0.00083, whereas inter-micro-clade diversity was 0.001 (Table S2). Coefficient of differentiation of micro-clades against the entire virus diversity in Japan was 0.5466 (Table S2). Each viral sequence from the four well-known outbreak cases (Table S1) formed an individual micro-clade along with their epidemiological descendent; i.e. the Osaka/Kobe case and the Fukuoka case corresponded to the micro-clade 1 and 10, respectively. On the other hand, viral sequences from the post-marriage party, from the Funabashi school, and Shizuoka/759 were concentrated into micro-clade 4. Geographical proximity was not observed in micro-clades other than those of the four outbreak cases. Micro-clade 10 (the Fukuoka case) belonged to cluster 1, while most of the other micro-clades belonged to cluster 2. Micro-clades 1 (Osaka/Kobe cases), 11 (Niigata, Tochigi and Utsunomiya cases) and 12 (Okinawa cases) originated from clusters 1.2, 1.3 and 1.3, respectively.

**Figure 4:**
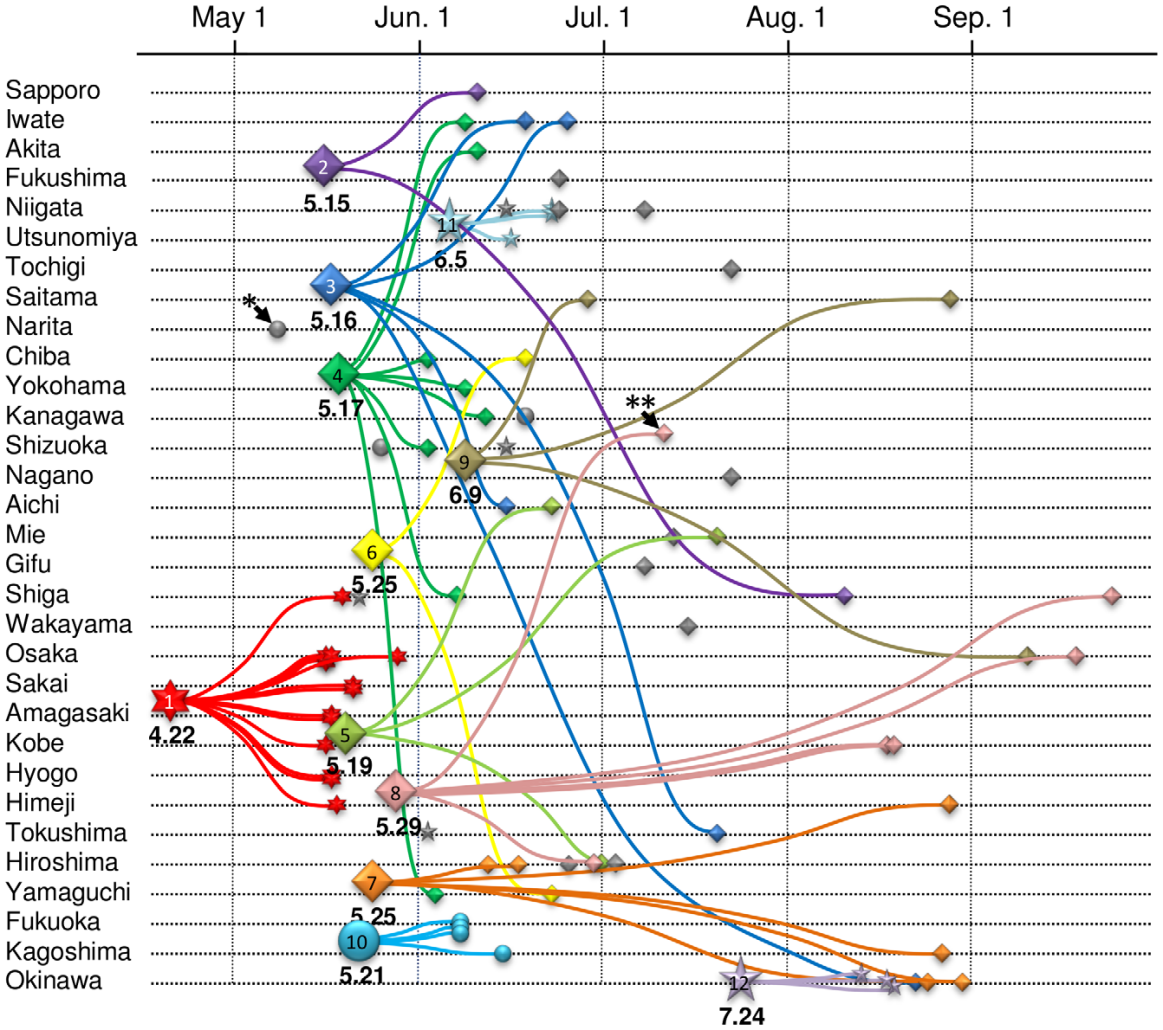
Schematic representation of the estimated profile of A(H1N1)pdm epidemic in Japan. The vertical and the horizontal axes show geographic localities and the times of viruses collection, respectively. The geographic localities are aligned in order of their latitude and the longitude as north-east to south-west. Seventy-five isolates of A(H1N1)pdm viruses from Japan are plotted by small symbols. The most recent common ancestor of twelve micro-clades inferred from the analysis are plotted by large symbol. Shape of the symbols indicate the cluster of each virus belonged; circle shows cluster 1, lozenge shows cluster 2, six-pointed star shows cluster 1.2 and five-pointed star shows cluster 1.3. The number below the large symbol shows tMRCA date. The number in the large symbol shows the micro-clade. The member of each micro-clade and their MRCA are linked with curves using different colors. The viruses which cannot classify any micro-clades are represented by gray symbols. A virus with a single asterisk is Narita/1 that was the first confirmed case in Japan. A virus with a double asterisk is A/Japan/PR1070 that had no epidemiological information.

Based on the Bayesian MCMC evolutionary estimates, tMRCA of micro-clade 1 (Osaka/Kobe cases) was dated to April 22, 2009 (April 11–May 1, Table 1). Micro-clade 12, consisting of viruses from only Okinawa had the latest tMRCA dated to July 24, 2009 (July 8–August 7, Table 1). tMRCAs of the other 10 micro-clades were ranged from May 15, 2009 to June 9, 2009 (Table 1). Figure 4 summarizes the tMRCA estimates of micro-clades, geographical and temporal properties of their members, and their phylogenetic relationships. The result demonstrated that most of the Japanese infection clusters except for Osaka/Kobe case emerged between late May and early June, 2009. Figure 4 also demonstrated that many MRCAs had disseminated their offspring over wide areas of Japan.

## Discussion

The A(H1N1)pdm viruses were shown to have diverged into distinct evolutionary clusters [12]. Our results on the evolution pattern of these viruses are in agreement with this previous study and provide additional information about the evolutionary relationship of the clusters. Data from our analysis elucidated an order within the cluster generation. tMRCA of cluster 1 preceded that of cluster 2 by two months, clearly indicating that viruses in cluster 1 were generated much earlier than other viruses. Phylogeny with swine influenza A viral segments as outgroups revealed that cluster 1 was a paraphyletic group of cluster 2, suggesting that the common ancestor of A(H1N1)pdm viruses was similar to cluster 1. The estimated tMRCA for all samples of A(H1N1)pdm was January 30, 2009, which is consistent with an previous study [11], and suggests that the first swine-to-human transmission of the A(H1N1)pdm occurred in January 2009 in Mexico and/or the US before the virus developed the cluster 1. On the other hand, tMRCA of cluster 2, at the beginning of April, 2009, coincides with the onset of second, larger outbreak in several communities including New York City schools [13]. The divergence pattern observed in the phylogeny demonstrated that cluster 1 was paraphyletic on cluster 2. A few variants in cluster 1 had therefore expanded in the New York outbreak, and generated the cluster 2 viruses. With a probable overlap in the infection areas of clusters 1 and 2, some co-infection cases may have induced the reassortment of viruses. It is likely that viruses in cluster 1.2 and 1.3 were derived from such a reassortant after the New York outbreak.

The chronological phylogeny also showed that the viruses belonging to cluster 2 seemed to be more successful in continuous circulation. They were widely distributed around the world, and found in more recently infected patients. Since only four amino-acid sites, which were not responsible for known viral phenotype [12], were variable in the cluster, the advantage of virus circulation of cluster 2 viruses is difficult to understand. Tajima's neutrality test showed that these variable amino-acid as well as the nucleotide substitutions were undergoing neutral or purifying selection. Epidemiological circumstances may therefore allow cluster 2 to dominate world-wide. The world-wide spread of reassortant strains could also be attributed to an epidemiological factor, although cluster 1.2 had a great impact on the Osaka/Kobe area in Japan.

We found 12 micro-clades that appeared to correspond to a particular case of infection cluster in Japan. Our criteria will purify the micro-clade by following steps. Step 1 and 2 determine some reliable monophyletic groups in the world-wide phylogenetic relationship of the viruses. Among these groups, micro-clades having an MRCA being directly associated with a virus from the other country (particular transported case to Japan) are selected at step 3. By the use of the criteria, we found some associations between the viruses in certain micro-clades and patients in well-defined outbreak cases in Japan (Table S1). In addition, degree of a differentiation of the micro-clade accounted for approximately 55% of total diversity of viral sequences in Japan (Table S2). These results suggest a validity of our criteria for inferring an infection cluster in Japan. Sixteen viruses did not belong to any micro-clades. Further analysis of additional isolates may allow these 16 unique viruses to be classified into novel micro-clades. Anyway, a total of at least 28 independent transported cases occurred in Japan during 2009 pandemic. Bayesian MCMC evolutionary analysis revealed that viruses from the first outbreak in Japan (Osaka/Kobe), were derived from a common ancestor which circulated in late April. Epidemiological investigation by the Infectious Diseases Surveillance Center (IDSC) of NIID in Japan found an index case in a patient without travel history, on May 2, 2009 (personal communication). The tMRCA estimates of micro-clade 1 suggest that a few transmission steps had occurred between the index case and other patients of the Osaka/Kobe outbreak. The Bayesian analysis also revealed that a common ancestor of the Fukuoka outbreak had circulated in late May, 2009. The Fukuoka outbreak came to light when infection of a junior high school student was confirmed on June 6, 2009, about two weeks after the estimated date of the index case [6]. The first confirmed case in the Osaka/Kobe outbreak was also reported about two weeks later than tMRCA by the official surveillance system (May 5, 2009 in Kobe city). These results suggested that in spite of Japan's efforts to execute an efficient surveillance system, there was a delay of a few weeks in the detection of pandemic influenza transmission.

Since Japan is an island country, we occasionally assume we can control emerging infectious diseases from abroad by the use of a quarantine system. In fact, infections in the first pandemic wave of A(H1N1)pdm between April and May, 2009, seemed to be well controlled in Japan. Viruses of the Osaka/Kobe as well as the Fukuoka cases were epidemiologically confined to certain districts [5], [6], and also eradicated from the feature lineage in our analysis. Moreover, the first outbreak case consisted of the reassortant viruses between the primitive lineage and the New York lineage, which is likely to emerge late in the epidemic in US and Mexico. However, preventive actions seemed to fail in Japan in late May, 2009. Our results demonstrated that more than ten distinct viruses invaded Japan between late May and early June, 2009, coincident with the withdrawal of the quarantine defense line. Some of these lineages appeared to circulate throughout summer, and then spread nation-wide. The aggressive suspension of classes in schools [14], as well as quick feedback of data from epidemiological investigations to local governments likely contributed to the suppression of primary spread of the disease, and may be a factor in the low case fatality rate of pandemic H1N1 in Japan [14]. In this context, it is noteworthy that the sequences from the other three well-characterized cases were concentrated in one group in the phylogeny. Among these cases, the post-marriage party consisted of young, active adults and did not include any school students, and the Funabashi case was not exactly a school cluster because patients spread the virus during a school excursion. Linkage of these cases suggests a vulnerability of certain communities, especially young adults, and that survival of virus leakages in such a community created a second epidemic wave in Japan between summer and autumn.

Control of the spread of infectious diseases requires a good grasp of a linkage among disease cases. While field epidemiologists generally identify the epidemiological linkage, they may sometime overlook certain cluster infections. We demonstrated that a virus belonging to a lineage other than the Osaka/Kobe case was detected in the Shiga prefecture during the first outbreak. Our reconstruction of an evolutionary relationship between viruses provides precise information about case linkages. Studies elucidating the association of an amino-acid change within a functional site and/or an accumulation of the genetic diversity will provide valuable information in the treatment and vaccine strategy of pandemic influenza.

## Materials and Methods

### Ethical statement

This study was conducted according to the principles expressed in the Declaration of Helsinki. The study was approved by the human subjects committee at the National Institute of Infectious Diseases, Japan. All patients provided written informed consent for the collection of samples and subsequent analysis.

### Influenza A(H1N1) viral gene sequences

A total of 74 A(H1N1)pdm viruses were collected and isolated at the National Institute of Infectious Diseases (NIID) or jurisdictional public health institutes in Japan between May 8, 2009, and September 22, 2009. Figure 1 shows the geographical location of the collection sites of the isolates. Known epidemiological linkages of patients carrying the viruses are shown in Table S1. Eight gene segments of the viruses were sequenced in NIID or National Institute of Technology and Evaluation, Japan. One hundred and sixty-three full-length nucleotide sequences from isolates collected world-wide, except for Japan, with known collection dates, were retrieved from the GenBank database. A full-length sequence of A/Japan/PR1070/2009(H1N1), whose epidemiological information was unknown but apparently derived from Japan, was also retrieved from the database. We also retrieved PB2-PB1-PA-HA-NP-NS sequences of four European avian-like swine H1N1 viruses and the NA-MP sequence of two triple-reassortant swine H1N2 viruses as origins of A(H1N1)pdm viruses [11]. The sequence list with the relevant accession numbers is available in supporting information (Table S3). The sequences were filed with each segment separately, and aligned using CLUSTAL W, version 2.0.10 [15]. The multiple alignment of each segment sequence was then reviewed manually to ensure that gaps did not alter the reading frame. We concatenated the segment alignments into a full-length, PB2-PB1-PA-HA-NP-NS, and/or NA-MP according to the outgroup composition.

### Distance-based phylogenetic inference

The sequence alignment of each segment, as well as the full-length alignment, was subjected to neighbor-joining (NJ) tree computation. This used 1000 bootstrap replicates from the matrix of the number of base substitutions per site based on the Maximum Composite Likelihood method [16]. All positions containing gaps and missing data were eliminated from the dataset. The mean number of base substitution per site (i.e. genetic diversity) within and between subpopulations were calculated on the basis of the distance matrix of 238 and 75 sequences for entire world and Japan, respectively. Standard error estimates were obtained by a bootstrap procedure with 500 replicates. Tajima's neutrality test [17] was also performed by full-length alignment data. The distance matrixes, the Tajima's test statistic, and the neighbor-joining trees were conducted using MEGA version 4.0 [18].

### Bayesian Coalescent MCMC evolutionary analysis

Evolutionary rates, molecular clock phylogenies, and other evolutionary parameters were estimated from heterochronous data of the full-length concatenated gene sequences of A(H1N1)pdm, using the Bayesian Markov chain Monte Carlo (MCMC) method. The nucleotide substitution model used in the analyses was evaluated by the hierarchical likelihood ratio test using PAUP v4.0 [19] with MrModeltest [20]. The general time-reversible (GTR) model with invariant sites (I) with four rate categories had maximum likelihood. Bayesian MCMC analyses were performed by BEAST v1.4.8 [21] using the substitution model of GTR+I, three partitions into codon positions, and a relaxed molecular clock model (the uncorrelated lognormal-distributed model) [22]. Three different population dynamic models (Exponential growth, Logistic growth, Constant population and Bayesian Skyline Plot (BSP)) were tested in the analyses. According to the BSP property, the logistic growth model, and the constant population model were adopted for the A(H1N1)pdm full-genome and swine influenza A partial genome, respectively. Each Bayesian MCMC analysis was run for 30 million states and sampled every 10,000 states. Posterior probabilities were calculated with a burn-in of 4 million states and checked for convergence with Tracer v1.4 [23]. The maximum clade credibility tree for analyzing the MCMC data set was annotated by TreeAnotator in the BEAST package. Detailed MCMC phylogeny with readable sequence name was shown in Figure S3. The posterior distribution of the substitution rate obtained from the heterochronous sequences was subsequently incorporated as a prior distribution for the mean evolutionary rate of the influenza A virus genome, thereby adding a timescale to the phylogenetic histories of the given viruses and enabling the times of the most recent common ancestors (tMRCA) to be estimated [24].

### Determination of “micro-clades” within the viruses circulating in Japan

In order to understand a viral lineage that was derived from a transported case to Japan, we determined monophyletic groups consisting of the Japanese viruses in the phylogeny. The group of viruses that fulfilled the following three criteria was defined as the micro-clade: (1) monophyletic group with above 75% bootstrap value on the distance-based tree (Figure S1), (2) monophyletic group on the maximum clade credibility tree of the coalescence-based analysis (Figure 2), and (3) viruses forming a monophyly or paraphyly of Japanese viruses in both trees. Involvement of one virus from other country was permissible. Each micro-clade was subjected to tMRCA estimation using BEAST.

## Supporting Information

Figure S1 Distance-based neighbor joining phylogeny of influenza A(H1N1)pdm full-genome sequences from around the world. The scale below the tree shows nucleotide substitutions per site. Numbers on the branch show bootstrap probabilities. Only >75% of the probability is shown in the figure. The clusters described in the previous report [12] are annotated by brackets on the right of the tree. The sequences from Japan are emphasized with bold type. Shadows on the terminal lineages show Japanese micro-clades. MC represents micro-clade.
(0.22 MB PDF)

Figure S2 Segment-divided neighbor joining phylogeny of influenza A(H1N1)pdm. Five trees inferred from the nucleotide alignment of HA, NP, NA, MP and NS are represented. Trees inferred from PA, PB1 and PB2 are not shown because no cluster divergence was observed in these segments. The clusters described in the previous report [12] are annotated by brackets on the right of the tree. Red and blue letters show the sequences derived from the cluster 1.2 and 1.3 viruses, respectively. Green letters show the other discordant sequences among the segmental trees.
(0.32 MB PDF)

Figure S3 Detailed presentation of Bayesian coalescence phylogeny of influenza A(H1N1)pdm full-genome sequence from the world. The branch length of the phylogeny is in units of time. The scale bar indicating 30 days of the branch length is drawn at the bottom of the tree. The clusters described in the previous report [12] are annotated by brackets on the right of the tree. Shadows on the terminal lineages show Japanese micro-clades. MC represents micro-clade.
(0.06 MB PDF)

Table S1 Epidemiological information about the outbreak cases and isolates in the A(H1N1)pdm epidemic in Japan.
(0.03 MB XLS)

Table S2 Estimates of the mean evolutionary diversity of A(H1N1)pdm in the world and Japan.
(0.02 MB XLS)

Table S3 Influenza A viral strains used in the study.
(0.71 MB XLS)
