# Supplementary figures and images for: Molecular Evolutionary Analysis of the Influenza A(H1N1)pdm, May–September, 2009: Temporal and Spatial Spreading Profile of the Viruses in Japan
Source: PLoS One. 2010 Jun 10;5(6):e11057. doi: 10.1371/journal.pone.0011057 (PMC2883557; doi:10.1371/journal.pone.0011057)

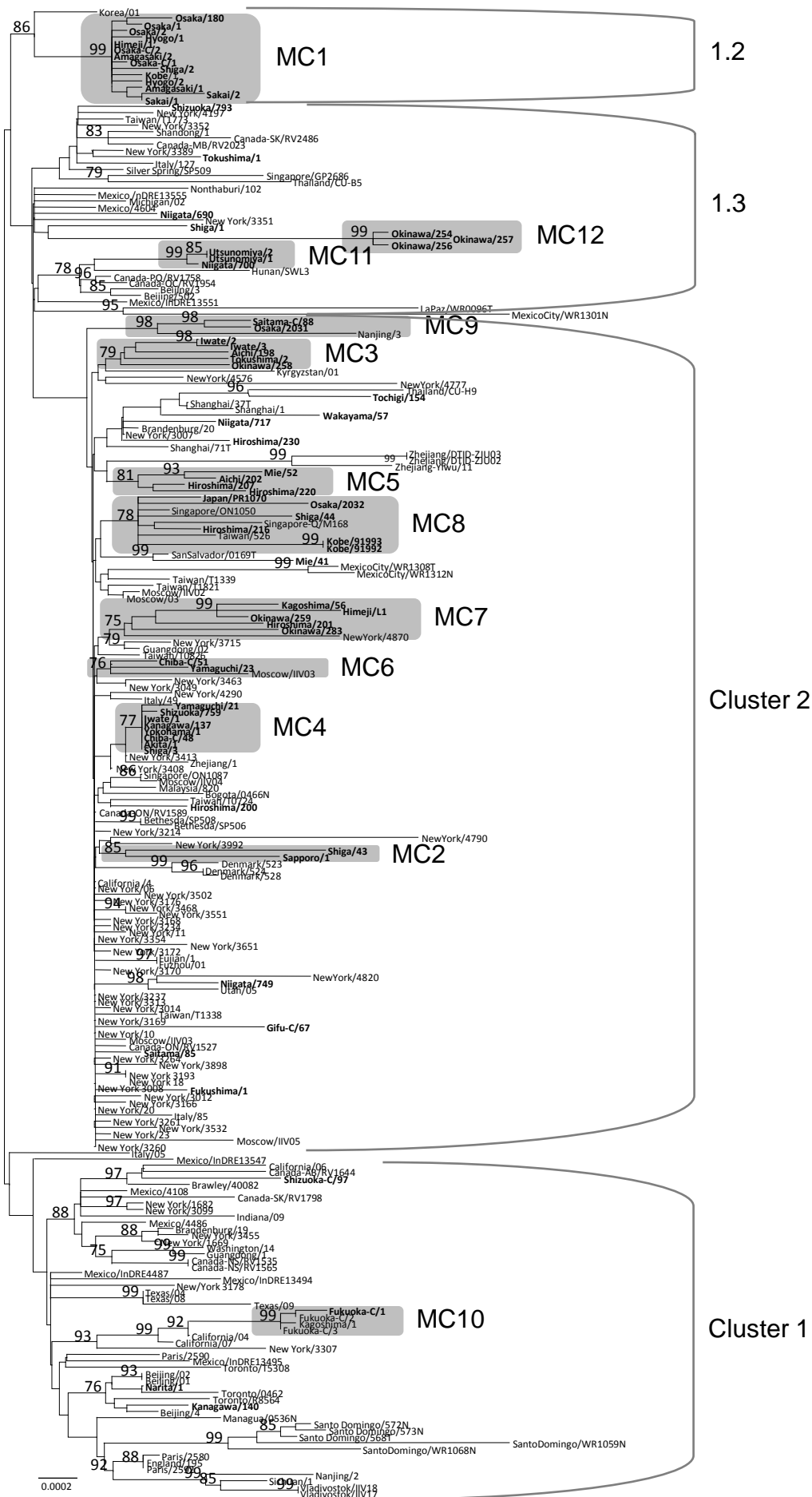

Supplement: Figure S1 — Distance-based neighbor joining phylogeny of influenza A(H1N1)pdm full-genome sequences from around the world. The scale below the tree shows nucleotide substitutions per site. Numbers on the branch show bootstrap probabilities. Only >75% of the probability is shown in the figure. The clusters described in the previous report [12] are annotated by brackets on the right of the tree. The sequences from Japan are emphasized with bold type. Shadows on the terminal lineages show Japanese micro-clades. MC represents micro-clade. (0.22 MB PDF) [file pone.0011057.s001.pdf]

HA

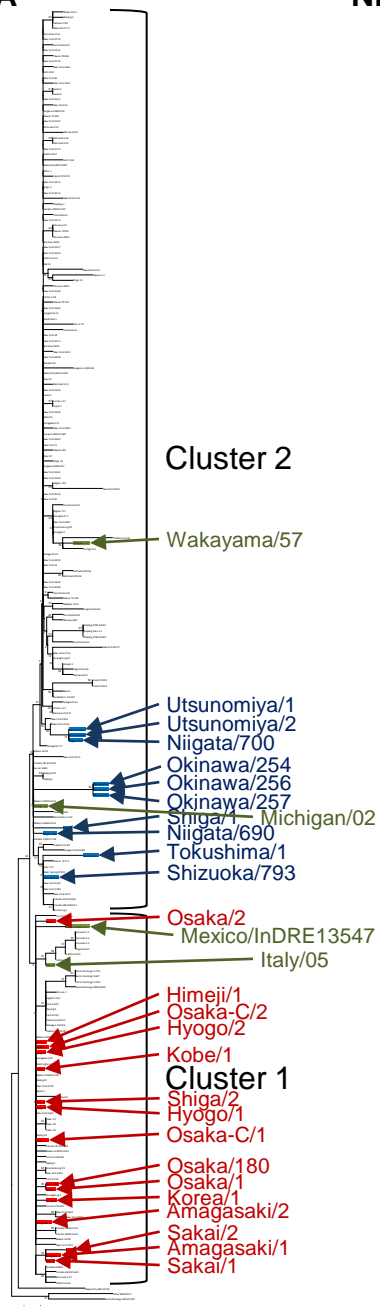

NP

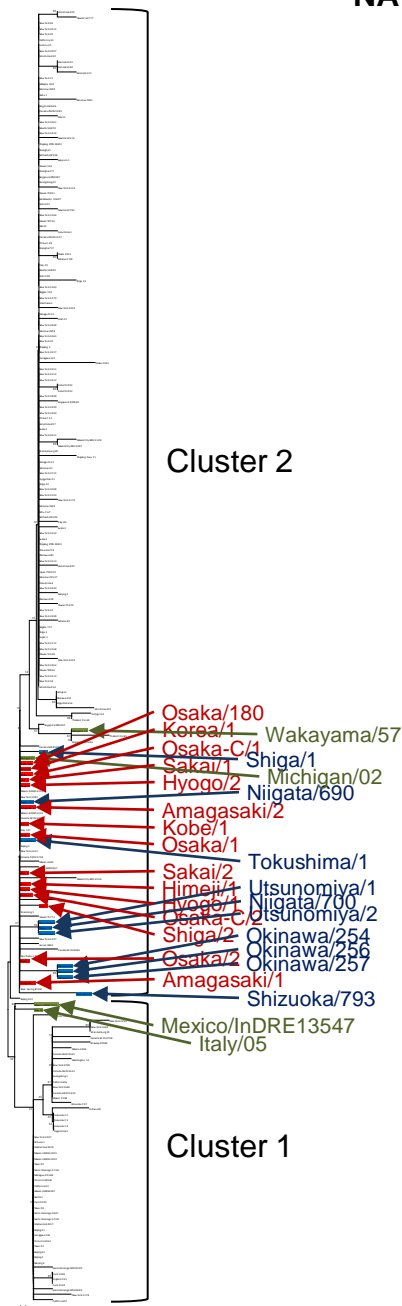

NA

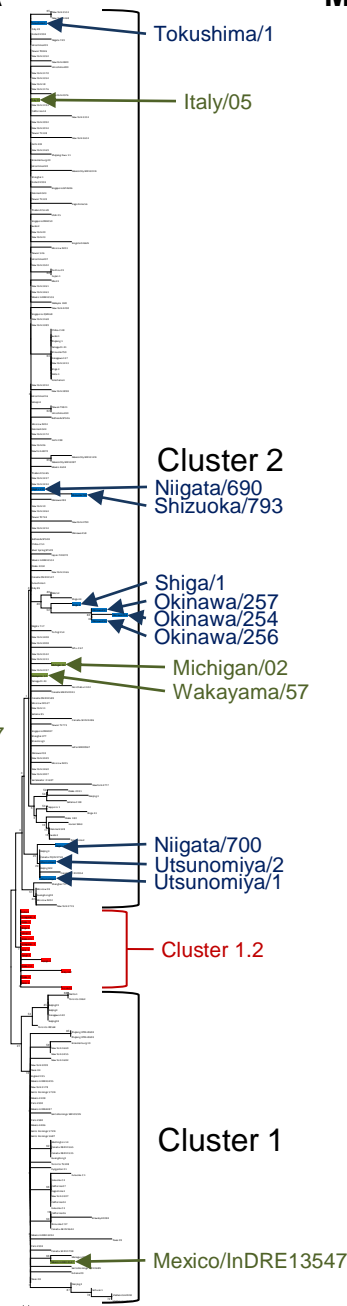

MP

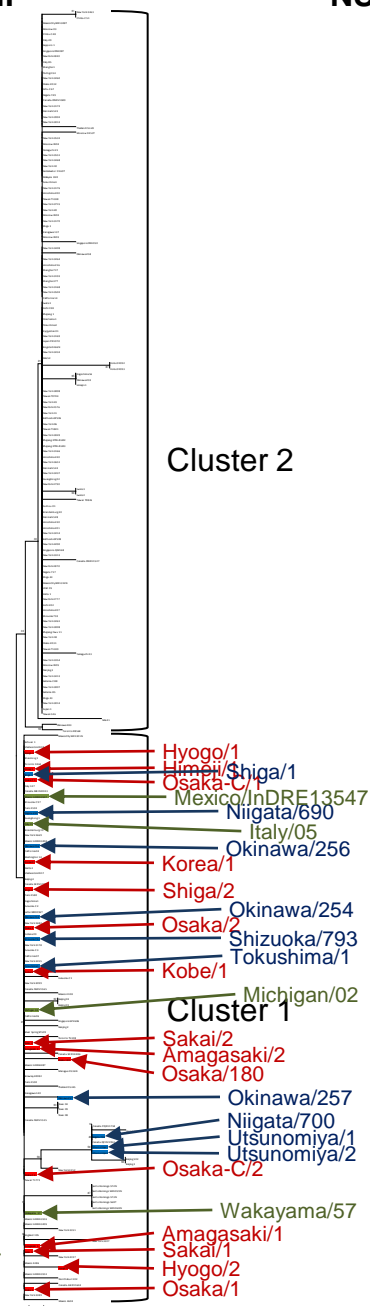

NS

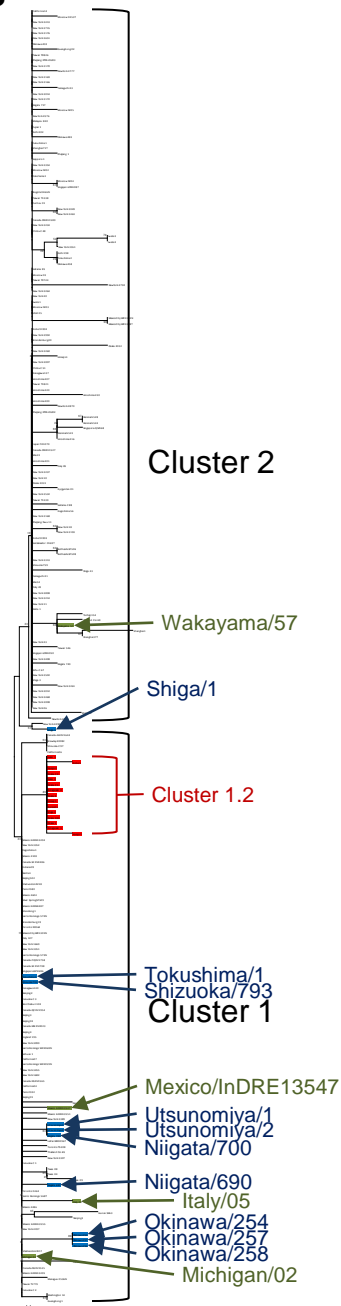

Supplement: Figure S2 — Segment-divided neighbor joining phylogeny of influenza A(H1N1)pdm. Five trees inferred from the nucleotide alignment of HA, NP, NA, MP and NS are represented. Trees inferred from PA, PB1 and PB2 are not shown because no cluster divergence was observed in these segments. The clusters described in the previous report [12] are annotated by brackets on the right of the tree. Red and blue letters show the sequences derived from the cluster 1.2 and 1.3 viruses, respectively. Green letters show the other discordant sequences among the segmental trees. (0.32 MB PDF) [file pone.0011057.s002.pdf]

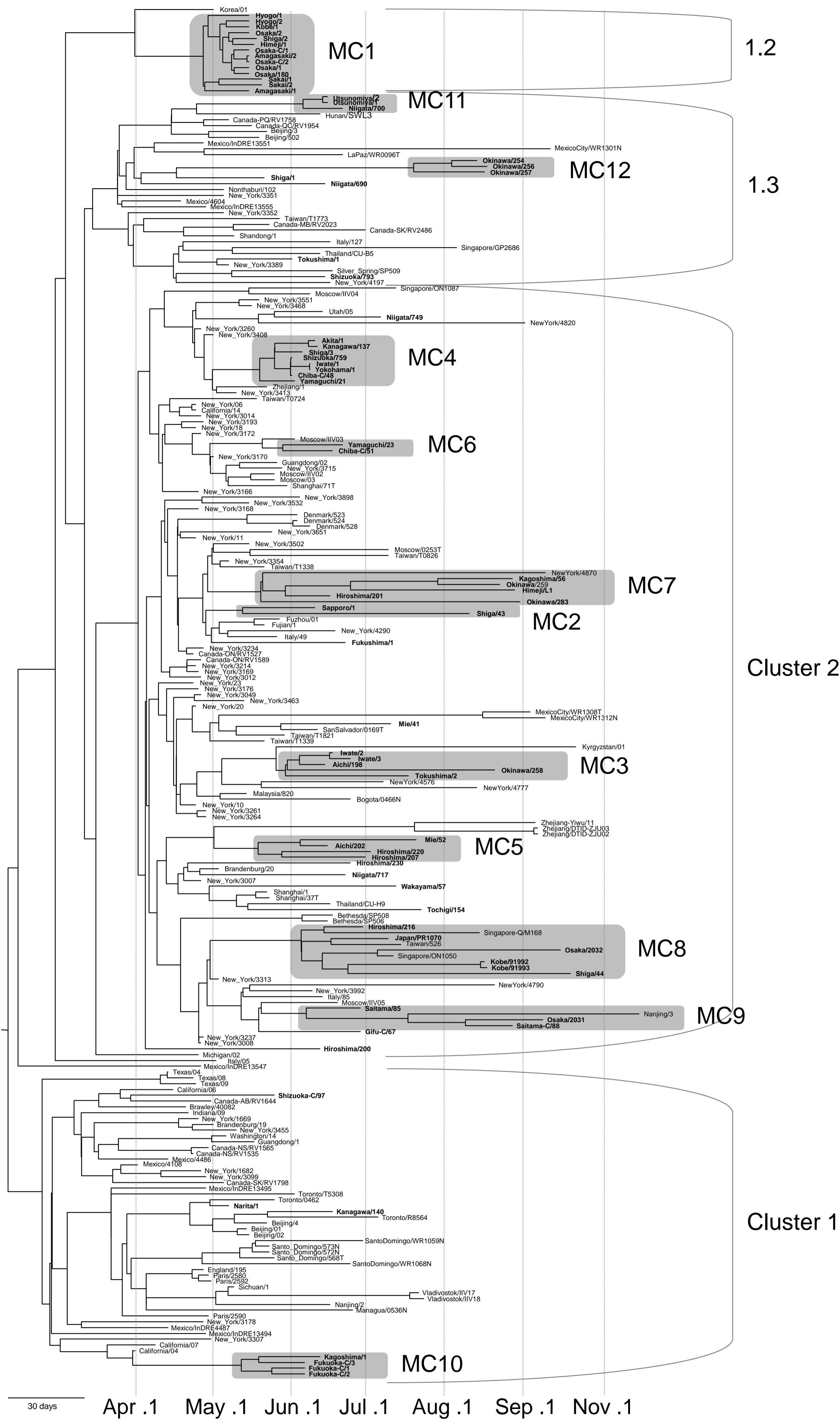

Supplement: Figure S3 — Detailed presentation of Bayesian coalescence phylogeny of influenza A(H1N1)pdm full-genome sequence from the world. The branch length of the phylogeny is in units of time. The scale bar indicating 30 days of the branch length is drawn at the bottom of the tree. The clusters described in the previous report [12] are annotated by brackets on the right of the tree. Shadows on the terminal lineages show Japanese micro-clades. MC represents micro-clade. (0.06 MB PDF) [file pone.0011057.s003.pdf]
